# *N*-Alkylated Chitin Nanocrystals as a Collector in Malachite Flotation

**DOI:** 10.1021/acssuschemeng.2c01978

**Published:** 2022-08-04

**Authors:** Robert Hartmann, Marco Beaumont, Eva Pasquie, Thomas Rosenau, Rodrigo Serna-Guerrero

**Affiliations:** † Department of Chemical and Metallurgical Engineering, School of Chemical Engineering, Aalto University, P.O. Box 12200, FIN-00076 Espoo, Finland; ‡ Fraunhofer Center for Chemical-Biotechnological Processes, D-06237 Leuna, Germany; § Department of Chemistry, Institute for Chemistry of Renewable Resources, University of Natural Resources and Life Science, A-3430 Tulln, Austria; ∥ Department of Bioproducts and Biosystems, School of Chemical Engineering, Aalto University, FIN-00076 Espoo, Finland; ⊥ Université Grenoble Alpes, CNRS, Grenoble INP (Institute of Engineering), LGP2, F-38000 Grenoble, France

**Keywords:** Chitin nanocrystals, Functionalization, Interfacial behavior, Hydrophobicity, Flotation

## Abstract

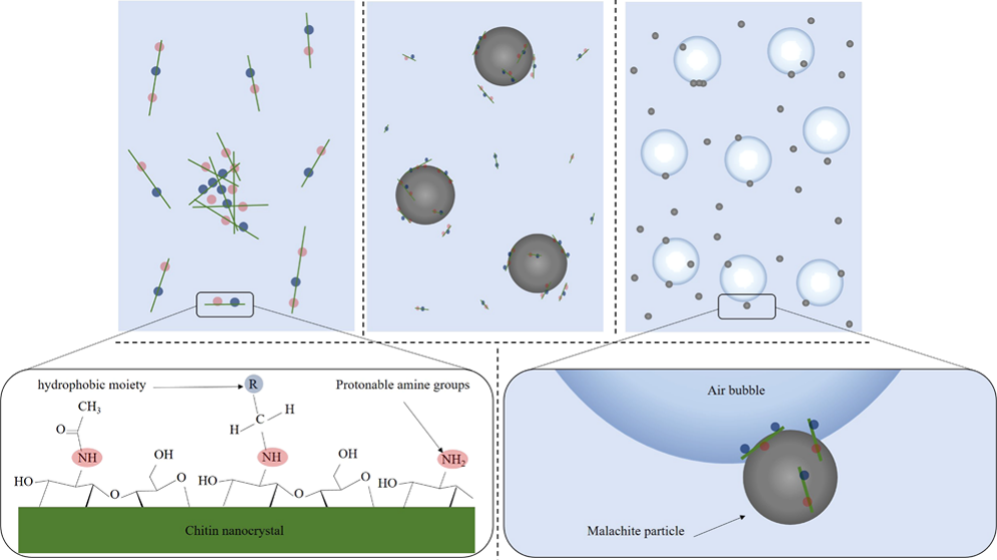

The majority of reagents currently used in mineral flotation processes are fossil-based and potentially harmful to the environment. Therefore, it is necessary to find environmentally-friendly alternatives to reduce the impact of mineral processing activities. Chitin nanocrystals are a renewable resource that, due to the natural presence of amino groups on its surface, represents a promising collector for various minerals of economic relevance. This study examines the one-pot functionalization of chitin nanocrystals with aldehyde structures to obtain hydrophobized colloids suitable for mineral flotation. The chemical properties of these nano-colloids were investigated by nuclear magnetic resonance spectroscopy, their colloidal behavior and structure by electrophoretic light scattering and atomic force microscopy, and their wettability through water contact angle measurements. The functionalized *N*-alkylated chitin nanocrystals possessed a hydrophobic character, were able to dress mineral particles and featured a performance in the flotation of malachite similar to commercial collectors, which proves the high potential of chitin nanocrystals in this field of application.

## Abstract 2

The use of functionalized chitin nanocrystals with integrated mineralophilic and hydrophobic moieties as a collector reagent for the flotation of malachite.

## Introduction

Flotation is the dominant industrial operation for the beneficiation of mineral resources, especially for finely dispersed minerals containing metallic elements, such as Cu, Co, and Li, which are relevant for continuously growing markets, for instance in electromobility or information technology.^1^ Like most industries, mining operations are searching ways to minimize their environmental impact, for instance by shifting from fossil to renewable-based resources. A major challenge in this transition is the identification of alternative raw materials, which also meet the requirements in terms of performance and cost-efficiency. Among the potential alternatives to oil-based chemicals, biopolymers offer attractive mechanical, chemical, and interface properties and, in many instances, they can be functionalized to tailor their properties toward specific applications. The most common biopolymers are cellulose and lignin, obtained mostly from plants, and chitin (from Old Greek χιτων - suit of armor), extracted from arthropods, mostly from the exoskeleton of insects and the shell of crustaceans, such as shrimps and crabs, although fungi are another potential source.^2^ The literature offers examples of disintegration and purification of these biopolymers and their applications as emulsifiers, interface stabilizers, coatings, membranes, and composites.^3−8^ Among those cases, nanocellulose has been a prime subject of many studies aiming at sustainable functional materials.^9,10^ In contrast, the production and use of chitin nanocrystals have remained largely underexplored.^11^ The lower interest in chitin may be related to its more difficult extraction and purification and its less obvious prevalence compared to cellulose, which has a long history of use in the pulp and paper industries and has thus become a mainstay in many national economies. Nevertheless, within certain applications, the presence of amino groups, naturally occurring in chitin, may represent an advantage due to their high reactivity,^10^ so chitin has also recently gained the interest of researchers searching for alternatives to fossil-based materials. The amino groups in chitin can act as a nucleophile and a base^12^ or as chemical anchors to attach other functional groups onto the chitin surface.^13^ In chitin’s “native state”, most amino groups are acetylated. A deacetylation pretreatment is required to cleave the acetamides and to increase the availability of the free primary amino groups (i.e., 2-deoxy-2-amino-β-d-glucopyranoside units).^14^ To obtain chitin nanocrystals (ChNCs), only the acetamides at the surface are hydrolyzed, whereas under more severe conditions (degree of deacetylation > 50%), chitin is transformed into the water–soluble biopolymer chitosan. Usually, deacetylation is performed under alkaline conditions. If followed by acidic hydrolysis of the amorphous components and subsequent sonication treatment, the procedure affords individualized spindle-shaped ChNCs.^15,16^ ChNC suspensions are colloidally stable under acidic to neutral conditions since in this range amino groups are protonated to electrostatically repulsive ammonium functions. The size and number of available amino groups at the surface of ChNC can be adjusted by the severity of the deacetylation process conditions (e.g., temperature, reactant concentration, time).^17,18^

To address the fossils-to-renewables transition in the mineral processing industry, some groups have started to explore sustainable alternatives to state-of-the-art reagents used in froth flotation.^19−22^ Simultaneously, flotation reagents are facing challenges with respect to their environmental impact and efficiency, due to strict regulations and ore depletion, respectively.^23^ To the best knowledge of the authors, a systematic study on the application of chitin-based reagents in flotation processes has not been reported so far. Literature only reported tests on chitosan (i.e., deacetylated chitin) as a flotation depressant that selectively adsorbed on mineral surfaces and retained their hydrophilic character to promote their dispersion in aqueous slurries. Studies by Huang et al.^24,25^ investigated chitosan as a selective depressant for chalcopyrite or sphalerite in mixtures with galena to replace hazardous inorganic depressants, such as cyanide, dichromate, or sulfur dioxide. Although chitosan depressed all three minerals in single-mineral flotation tests, it showcased selectivity toward chalcopyrite in mixed mineral systems, leading to 65% higher recoveries of galena compared to chalcopyrite. Similarly, sphalerite was selectively depressed by chitosan in mixed mineral systems with galena, when either ethylenediaminetetraacetic acid was added to the flotation slurry or when sphalerite was coated with Cu^2+^-ions beforehand. In a more recent study, the depression of talc by chitosan was reported.^26^ While these studies emphasize the potential of chitosan to be used as depressants, they also exemplify that a proper understanding of the complex interaction between chitosan and mineral species is a prerequisite to attaining adequate performance.

Although the chemical composition of ChNCs and chitosan is similar (see Figure S1), conceding differences in the amide/amine ratio, their behavior in flotation processes may significantly differ due to the colloidal state of ChNCs. In contrast to ChNCs, chitosan is a linear, water–soluble polysaccharide.^10^ Most traditional flotation reagents are either water–soluble molecules of moderate sizes, such as amines,^27^ xanthates,^28,29^ fatty acids,^30^ soy bean oil,^31^ hydroxamates,^32^ or macromolecules, e.g., chitosan,^24,25^ and starch.^33^ The latter are usually not further functionalized when used as depressants due to their inherent hydrophilicity and the resulting wetting properties. Water–soluble collector molecules possess a relatively simple amphiphilic structure, containing a hydrophobic component, usually a hydrocarbon alkyl chain on the one end and a functional group interacting with the mineral surface on the other. In contrast to depressants, collectors are applied to render the surface wettability of selected mineral species more hydrophobic to promote orthokinetic particle-bubble attachments and thus their enrichment in the flotation froth. Owing to their colloidal state, natural nano-colloids exhibit different interaction mechanisms when used in flotation processes. This interaction of colloidal collectors was first studied using artificial polymer nanoparticles,^34−36^ which, however, had insufficient colloidal stability in the flotation slurry^37^ and showed detachment from the mineral surface under turbulent conditions.^38^ Moreover, the employment of nanoparticles led to previously unknown phenomena, such as the occurrence of wet-patch adhesion,^34^ where hydrophobic nanoparticles adsorbed onto mineral particles attach to air bubbles, while the mineral surface remains completely wetted by water. Nevertheless, high flotation recoveries have been obtained with these systems,^39^ but these artificial, oil-based polymer nanoparticles do not represent sustainable alternatives to conventional reagents. Based on these findings, a cellulose-based reagent, namely aminated cellulose nanocrystals (ACNCs), was successfully used for the recovery of quartz.^19,21^ According to studies of the ACNC interaction with the mineral surface, their insolubility in water causes fundamentally different action modes for their dispersion, attachment on the mineral surface, and subsequent formation of orthokinetic particle-bubble aggregates.^22,40^ The aqueous medium defines the activity of the amino groups in ACNCs and thereby affects the interactions between individual nanocrystals, nanocrystals and mineral surfaces, as well as nanocrystals and air bubbles.^41^ Reportedly, using adequate surface modification and optimized slurry conditions, ACNCs can improve the recovery of quartz compared to conventional, amphiphilic collectors.^22^

The preparation of ACNCs involves an oxidation treatment with sodium periodate to introduce aldehyde groups, which enables in a subsequent reaction the introduction of hydrophobic amines onto their surface.^42^ In contrast, partially deacetylated ChNCs naturally possess amino groups at their surface. The amino groups can be protonated to introduce stability in aqueous colloidal dispersions (through repulsive positive surface charges) and enable electrostatic interactions with the negatively charged mineral surfaces, as well. In addition, the amino groups enable further functionalization, for instance through reaction with aldehydes.

This study investigates the properties of functionalized ChNCs with an outlook on their application as mineral processing reagents. Furthermore, this work explores their use in the flotation of malachite, a basic copper carbonate as a case study. The choice of malachite in this study corresponds to its increasingly significant role as a resource of copper due to the overexploitation of copper sulfide minerals. The annual copper demand in 2020 has been 26.4 million tons of which around 30% have been extracted from copper oxide ores.^43,44^ Therefore, flotation represents a remarkable market for chitin-based reagents that have an annual production of 100 gigatons in the biosphere,^45^ of which 2.3 million tons are produced annually by crustaceans.^46^ However, a major challenge for the exploitation of malachite is its unresponsiveness to traditional copper sulfide collectors, such as xanthates,^47^ fatty amine derivates,^43^ or hydroxamates.^32^ Therefore, artificial talc nanoparticles,^48^ alternative flotation schemes, or the sulfidation of malachite before collector adsorption have been considered^49,50^ as alternative approaches to improve malachite recovery. As a novel approach, this work examines the functionalization of partly deacetylated chitin nanocrystals with aldehydes for the direct flotation of malachite.

## Experimental Section

### Materials

Chitin powder extracted from shrimp shells was purchased from Sigma-Aldrich (Product No. C7170). To remove residual inorganic contaminants (mostly carbonates), chitin (10 g) was dispersed in 250 mL of a 1 M HCl solution at 85 °C for 3 h. The suspension was allowed to cool down and chitin was washed six times with purified water (Milli-Q water, 18.2 MΩ cm) using a centrifuge at 2500 g acceleration (Thermo Scientific, Megafuge 16R). The purified chitin was dispersed in 250 mL of a 12.4 M NaOH solution at 95 °C for 4 h for deacetylation, washed three times by centrifugation, and dialyzed (cellulose membrane, *M*_w_ cutoff = 14 kDa) against Milli-Q water. The sample thus obtained was dispersed in 250 mL of a 3 M aqueous HCl solution at 85 °C for 2 h for acidic hydrolysis. The obtained chitin nanocrystals (ChNCs) were washed after deacetylation and treated with a titanium-tip sonicator (Sonifier 450, Branson Ultrasonics Co., Danbury, CT) at 200 W (50% power) for 1 min using 10 s on-off cycles and stored at 4 °C in a refrigerator. The degree of acetylation was 89%, determined by conductometric titration, and ChNCs featured a positive surface charge with 0.574 mmol g^–1^ of amino groups.

Malachite rocks (Ward’s Science) were crushed in a jaw crusher to obtain particles with a maximum size of 3 mm. In a second milling step, 70 g of malachite were treated for 15 s in a ring mill (Fritsch PULVERISETTE, planetary micro mill) using a tungsten carbide ring. The milled sample was dry-sieved using a 150 μm mesh for 30 min. The undersize fraction was dispersed in purified water and sonicated for 3 min before being placed on a 20 μm sieve and washed with Milli-Q water. The remainder fraction was repeatedly sonicated and sieved until the water passing the sieve appeared clear, suggesting the removal of fines. The obtained particle size distribution was determined on a Malvern Mastersizer 3000 (shown with size quantiles in Figure S2). To prepare an ultrafine fraction of malachite (*x* < 1 μm), 25 g of the crushed malachite sample (*x* < 3 mm) were further treated in the ring mill using 6 cycles of 15 s each.

### Functionalization of ChNCs

**Figure 1:**
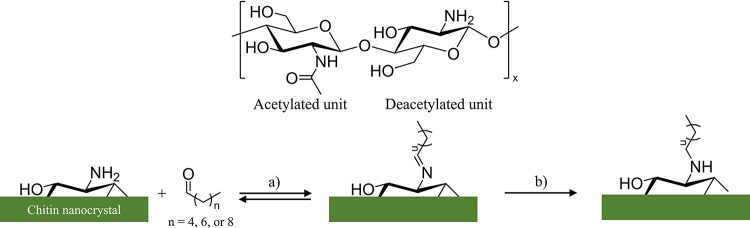
Partly deacetylated chitin (top) and its reductive alkylation by aldehydes of different chain lengths (bottom, hexanal *n* = 4, octanal *n* = 6, or decanal *n* = 8): (a) reversible imine bond formation and (b) irreversible imine reduction by an α-picoline-borane complex.

Aliphatic aldehydes with different chain lengths, namely hexanal, octanal, and decanal, were purchased from Sigma-Aldrich with a purity ≥ 98%. ChNC functionalization with aldehydes was performed in 0.1 M acetic acid (≥98%, VWR, pH 4.5). The aldehyde (fivefold molar excess relative to the amino groups) was dissolved in 2-propanol (10 mL, Sigma-Aldrich) before addition to a ChNCs suspension (0.2 g absolute dry weight per sample) in water. α-picoline-borane complex (95%, Sigma-Aldrich, tenfold molar excess relative to the amino groups) was dissolved in 0.1 M aqueous acetic acid under heating to 70 °C and added to the reaction mixture to convert the initially formed labile azomethine bonds into stable C–N bonds (reductive amination of the aldehydes). The reaction time for the samples was set to either 3 h, 3 days, or 7 days and was performed under ambient temperature (22 °C). A fourth sample was prepared to allow the reaction to take place for 3 h at 80 °C. The reaction scheme is shown in Figure 1. After the reaction, the suspension of functionalized ChNCs was dialyzed overnight against a Milli-Q water/ethanol mixture (4:1, v/v) and then against Milli-Q water for 5 days, replacing the medium twice a day. The purified samples were stored in a fridge at 4 °C.

### Characterization of Chemical Composition

The degree of acetylation was calculated by conductometric titration on 0.5 g chitin (dry mass). The ChNCs were dispersed in 100 mL of Milli-Q water followed by the addition of 1 mL of 0.1 M aqueous HCl solution and 0.5 mL of 0.5 M aqueous NaCl solution. Using an automatic titrator (Metrohm) 0.1 M NaOH solution was dosed in 0.05 mL intervals under continuous magnetic stirring. The total volume of NaOH added between the two inflection points (*V*_equiv_) was used to calculate the degree of acetylation (DA) of the ChNCs suspension according to eq 1
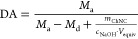
1where *c*_NaOH_ is the concentration of sodium hydroxide, *m*_ChNC_ is the absolute mass of chitin nanocrystals, and *M*_a_ and *M*_d_ are the molar masses of the acetylated (203 g mol^–1^) and deacetylated (161 g mol^–1^) chitin, respectively.^51^

Solid-state ^13^C NMR was used to characterize the structure of the extracted ChNCs. Before each measurement, the nanocrystal samples were hydrated in demineralized water for 24 h at room temperature, and excess water was removed with the aid of adsorbing paper. The NMR experiments were performed on a Bruker Avance III HD 400 spectrometer (resonance frequency of ^13^C of 100.61 MHz) equipped with a 4 mm dual broadband CP-MAS probe. The degree of acetylation was estimated following the procedure of Pasquier et al.,^52^ comparing the integrals of C2 (51–58 ppm region) and CH_3_ (20–25.5 ppm) peaks.

The degree of *N*-alkyl modification in the samples was estimated by relating the integral of the C1–C6 peaks (chitin polymer structure, 53–107 ppm region) to the peak region corresponding to the introduced alkyl chains and acetyl groups (12.5–34 ppm). The integral of the C1–C6 peal region was set to 6, and the integrals of the samples from 12.5 to 34 ppm were defined as *I*_sample_ (see Figure S3–S7). With *I*_nat_ being the respective integral of the native, non-functionalized chitin sample, the degree of substitution (DS) was calculated according to eq 2.
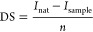
2where *n* is the number of carbons of the *N*-alkyl group.

### Structural Analysis and Electric Surface Potential

The size and shape of ChNCs were visualized by atomic force microscopy (AFM, Park Systems NX12). Glass slides (VWR microscope slide) used as sample holders were rinsed with copious amounts of water and sonicated three times for 3 min in Milli-Q water and propanol to remove any contaminant. For sample preparation, a ChNCs suspension of 0.1 wt % was diluted to 0.0001 wt % using ethanol (HPLC grade, Carl Roth), placed on the microscope glass slide, and allowed to dry under ambient temperature. Images were taken in non-contact mode and evaluated with the Gwyddion software (length and height of ChNCs).

The electrophoretic mobility of malachite and ChNC samples was measured with a Malvern Zetasizer Nano ZS90. For measurements of malachite, ultrafine malachite (10 mg, size < 4 μm) was dispersed in 10 mL of a 10 mM NaCl background solution at pH 3, 5, 7, or 9 and, after 2 min of conditioning, was transferred into a disposable cuvette. The electric ζ-potential was determined six times. In the case of ChNCs, the suspension (100 μL, 0.1 wt %) was added to 10 mL of background solution and the same routine was applied as to malachite. To analyze the effect of ChNCs dressing on the electric surface state of malachite, 10 mg of malachite were dispersed in the background solution and different volumes of a 0.1 wt % ChNC suspension were added. After 2 min of conditioning, the ζ-potential of ChNCs-coated malachite was determined.

### Surface Wettability and Flotation Performance

The degree of hydrophobicity of functionalized ChNCs was estimated through contact angle measurements using the sessile drop method (Theta optical tensiometer, Attension). Silicon wafers were cut into approximately 4 × 2 cm^2^ pieces, onto which a suspension of ChNCs (0.1 wt %, 500 μL) was placed and dried in a desiccator overnight. For contact angle measurements, a drop of Milli-Q water with a volume of 13 ± 1 μL was placed on the ChNC-coated silicon wafer, and the contour of the drop matched using the Young–Laplace equation. At least 6 contact angles were measured for each ChNCs sample.

The flotation experiments were performed in an in-house built Hallimond tube (dimensions shown in Figure S8) with a total volume of 150 mL. For conditioning, malachite (1 g) was dispersed in a background solution (10 mM NaCl, pH 3) for 2 min before ChNCs were added and stirred for 2 min. The sample was transferred into the Hallimond tube and the experiment started when the first bubbles entered the slurry. The suspension was agitated by a magnetic stirrer at a speed of 200 rpm, the air flow was set to 30 mL min^–1^ and the flotation time was 10 min. The overflow and underflow were collected and dried in a natural convection oven overnight at 50 °C before the recovered masses were determined. Additional experiments were performed using dodecylamine (DDA, Sigma-Aldrich) as a benchmark state-of-the-art collector, following identical conditioning and flotation procedures.

## Results and Discussion

For the targeted application of ChNCs as a mineral collector, contact angles of sessile drops on ChNC films were examined to preselect candidates that possess a sufficiently hydrophobic character. Subsequently, the chemical surface state of these candidates was further characterized to gain a better understanding of the behavior of ChNCs in flotation. Therefore, the influence of ChNCs on the electrostatic surface potential of malachite was investigated to infer their adsorption on the mineral surfaces. Flotation results were then compared with those obtained using dodecyl amine (DDA), a commercially available, water–soluble collector for malachite.

### Hydrophobicity of ChNC Films

**Figure 2:**
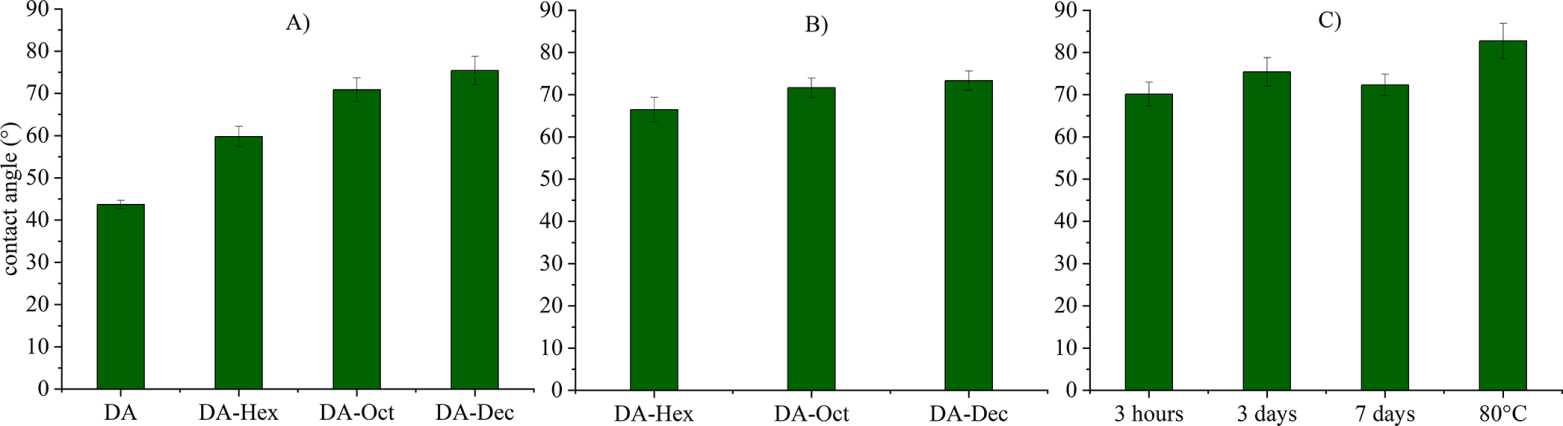
Three-phase contact angle of deacetylated ChNCs (DA) functionalized with *N*-hexyl (Hex), *N*-octyl (Oct), and *N*-decyl (Dec) moieties for (A) 3 days, (B) 7 days, and (C) ChNCs functionalized with decanal for different times and temperatures. The error bars represent standard deviations.

Sufficiently high hydrophobicity is a prerequisite to a successful use of ChNCs as a malachite collector. For estimation of the hydrophobicity changes of functionalized ChNCs, the three-phase contact angles of water droplets on ChNC films were determined, as shown in Figure 2.

In general, the contact angle increases proportionally to the length of the alkyl chain, while the reaction time during functionalization had only a moderate effect. This reflects the high reactivity between amines and aldehydes. The ChNC sample functionalized with decanal at 80 °C had the highest contact angle of 84° and thus the highest degree of hydrophobicity among the examined ChNC samples. The contact angles for ChNCs functionalized with *N*-decyl groups are similar to those for aminated cellulose nanocrystals previously used in the successful flotation of quartz.^40^ This level of hydrophobicity of *N*-decyl ChNCs was the optimum, which justified their choice for further studies on the chemical surface state, interactions with malachite and use in flotation processes.

### Physicochemical Characterization of *N*-Modified ChNCs

Different reaction conditions were tested for the reductive alkylation, which we discuss in the following section, comparing the influence of these conditions on the properties of ChNCs. It is important to consider that the functional ChNCs may still feature free amino groups, which are important for colloidal stability, as well as for electrostatic interactions with the mineral particles. The hydrophobic decyl groups might impact the dispersibility of ChNCs in aqueous media or partially shield the cationic charge due to hydrophobic interactions between nanocrystals.

**Figure 3:**
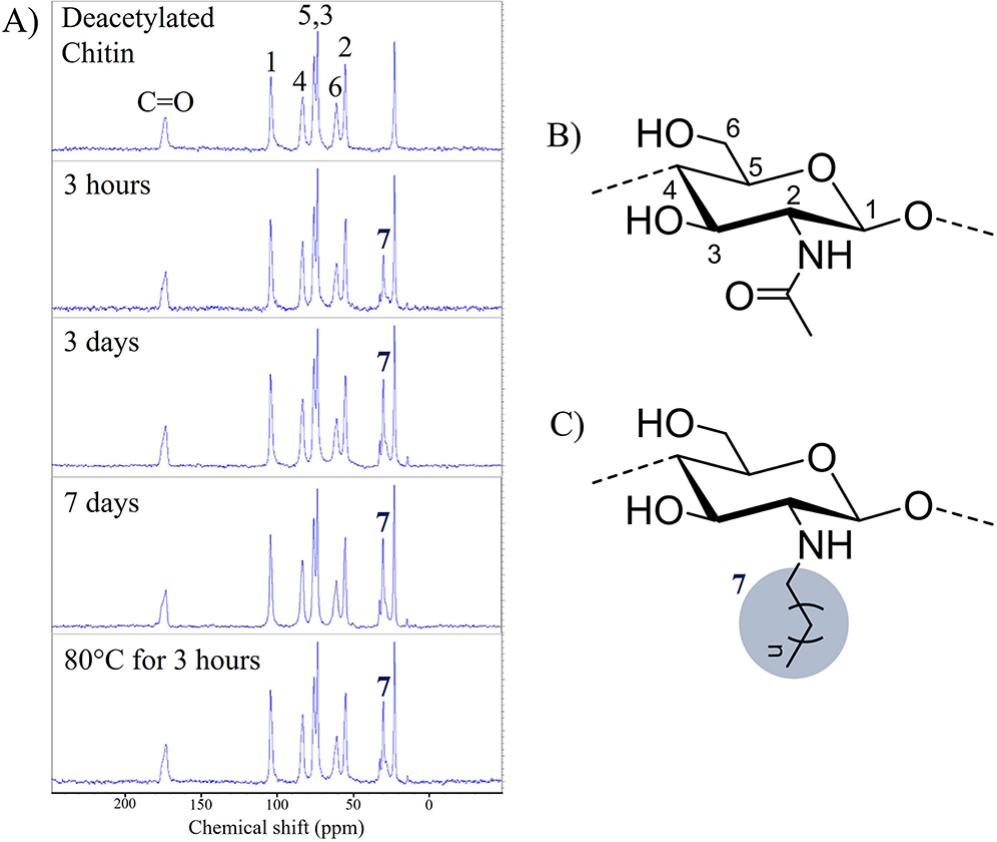
(A) Solid-state ^13^C NMR spectra of chitin nanocrystals with partial *N*-decyl modification (*n* = 8), introduced under different reaction conditions, in comparison to the native nanocrystalline sample. (B) Molecular structure and atom numbering of an *N*-acetylated monomer unit and (C) deacetylated, *N*-alkylated counterpart.

The solid-state ^13^C NMR spectra of natural ChNCs and ChNCs functionalized with decanal for different reaction times and temperatures are shown in Figure 3. These analyses were used to estimate the number of decyl groups introduced under different reaction conditions (Table 1).

**Table 1:** Substitution Degree of Decyl Groups Introduced Onto Chitin Nanocrystals Relative to the Total Number of Monomer Units in Dependence on the Conditions of the Reaction with Decanal/α-Picoline-Borane Complex^a^

sample	amount of *N*-decyl functionality (mol %)
native	
3 h	8.2
3 days	11.4
7 days	12.2
80 °C for 3 h	11.1

a Values estimated from solid-state ^13^C NMR results.

No impurities, such as proteins or reaction byproducts, were detected in the ChNCs by the ^13^C NMR spectra, proving that the prepared samples were of high purity within the detection limit of the method (∼3%). The number of amino groups available for modification in the native samples was 0.574 mmol g^–1^, measured by conductometric titration.

After reductive alkylation with the decanal/α-picoline-borane complex, new peaks around 30 ppm (methylene groups) and around 12 ppm (terminal methyl) were detected confirming the grafting of alkyl groups on ChNCs. The peak intensity correlated with the number of incorporated decyl groups and increased with reaction time from 3 h to 3 days.

Although the solid-state ^13^C NMR experiment is not considered a quantitative method to determine the concentration of species, a relative comparison of the number of decyl groups at the different ChNC sample surfaces is possible. An increase of the reaction temperature from ambient to 80 °C for 3 h reaction time introduced 35% more decyl groups at the ChNC surface. Similarly, extending the reaction time from 3 h to 3 and 7 days increased the *N*-decyl groups by 35 and 50%, respectively. Increasing the reaction temperature allows significantly faster incorporation of functional groups, although longer reaction times may be preferred in cases where energy costs are prohibitive.

**Figure 4:**
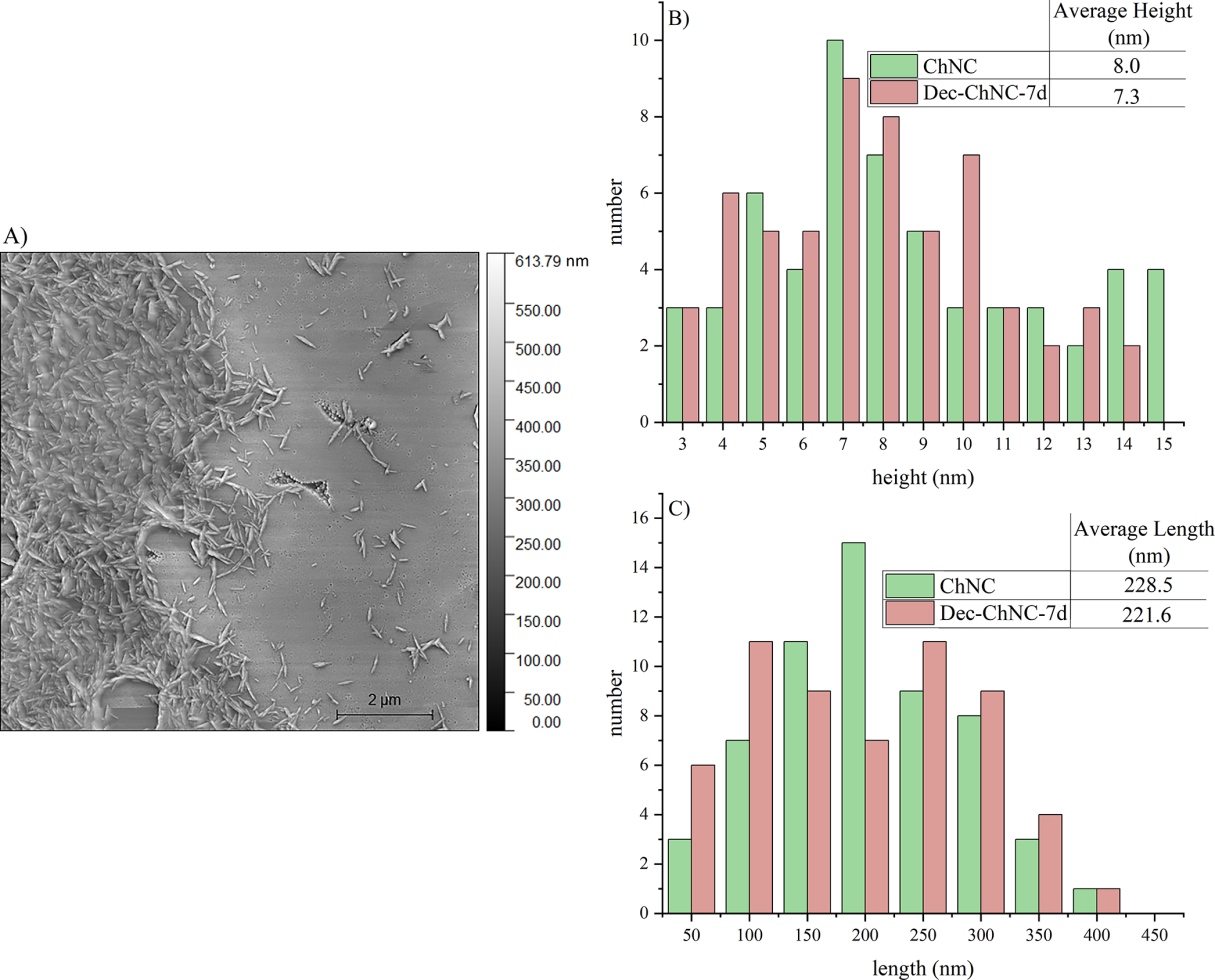
(A) AFM image of deacetylated chitin nanocrystals (ChNC); the distribution of the (B) height and the (C) length of deacetylated chitin nanocrystals and nanocrystals after the reaction with the decanal/α-picoline-borane complex for 7 days (Dec-ChNC-7d).

The effect of the reaction conditions on the shape and dimensions of ChNCs was visualized using AFM. A representative image and the obtained nanocrystal dimensions are presented in Figure 4.

The AFM images showed the typical rod-like structure of the nanocrystals with a width between 3 and 15 nm and a length between 50 and 400 nm. This is in accordance with typical dimensions for ChNCs reported in the literature, with lengths ranging from 150 to 500 nm and widths between 5 and 20 nm.^53^ As seen, the functionalization process did not significantly alter the dimensions of the ChNCs.

### Electric Surface Potential

**Figure 5:**
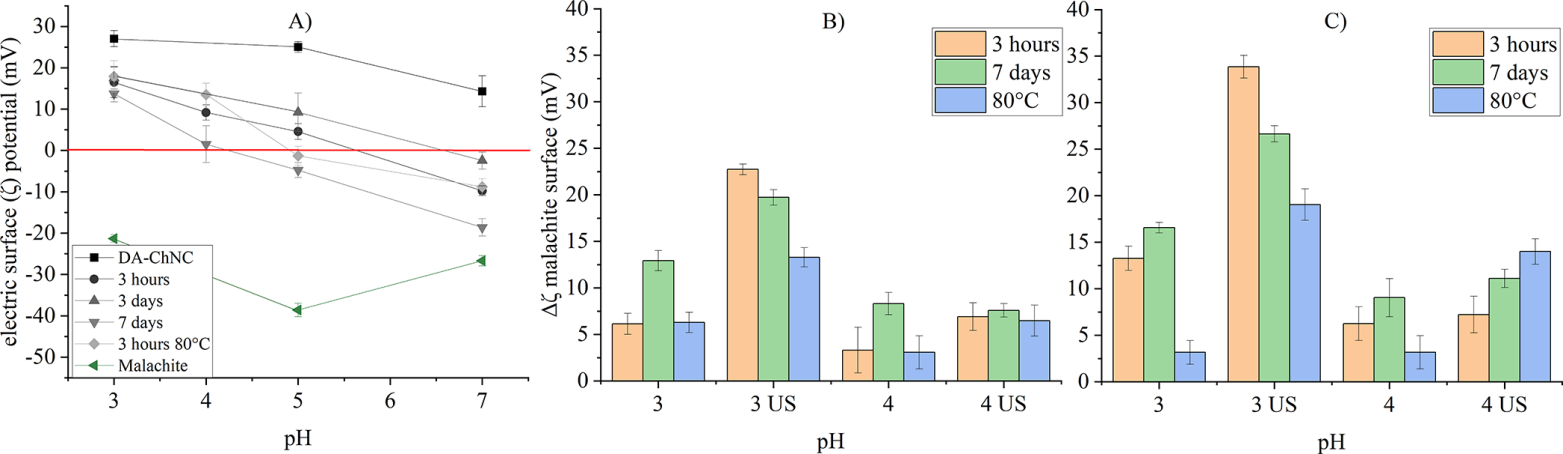
ζ-potential of deacetylated chitin nanocrystals (DA-ChNC), (A) ChNCs functionalized with *N*-decyl at different reaction times and temperatures and malachite; (B) change of the ζ-potential of malachite in the presence of 50 g kg^–1^ and (C) 100 g kg^–1^*N*-decyl ChNCs added directly or after ultrasound treatment (US) (right). The error bars represent standard deviations.

Similar to the hexyl-aminated cellulose used for the flotation of quartz, also in ChNCs the positive surface charges are expected to promote electrostatic attraction toward the malachite surface,^41^ resulting in a sufficiently strong adhesion on the mineral surface to withstand potential detachment and redispersion during the flotation process.^21,22^ The electric surface potential of the individual phases is shown in Figure 5 together with the net change of the malachite surface after coating with ChNCs.

The deacetylated ChNCs possess positive ζ-potentials over the examined pH range, while malachite presents negative charges. With the incorporation of decyl-groups on the ChNC surface, the ζ-potential of ChNCs was reduced, reaching an isoelectric point (IEP) at pH values between 4.5 and 6.5, depending on the functionalization conditions. The reduction of the ζ-potential originates from the reaction of the amines with the aldehydes, leading to limited accessibility of the amino groups for protonation and an increased tendency of flocculation due to higher hydrophobicity. To promote electrostatic attraction between functionalized ChNC samples and malachite due to opposite charges, pH 3 and 4 were chosen to investigate the adsorption process.

As seen in Figure 5, the ζ-potential of malachite was rendered more positive after mixing with ChNCs, corroborating the modification of the mineral surface. Simultaneously, sonication before conditioning with malachite leads to a better compensation of the negative ζ-potential of malachite compared with the untreated samples. This may be explained by the aggregation of ChNCs or the spontaneous organization of ChNCs into aggregates (chiral nematic structures^17^), leading to the formation of ChNC flocs (see Figures S9 and S10). By the ultrasound treatment, the well-dispersed state of ChNCs was restored and their attachment efficiency was improved.

### Floatability of Malachite Using Functionalized Chitin Nanocrystals

**Figure 6:**
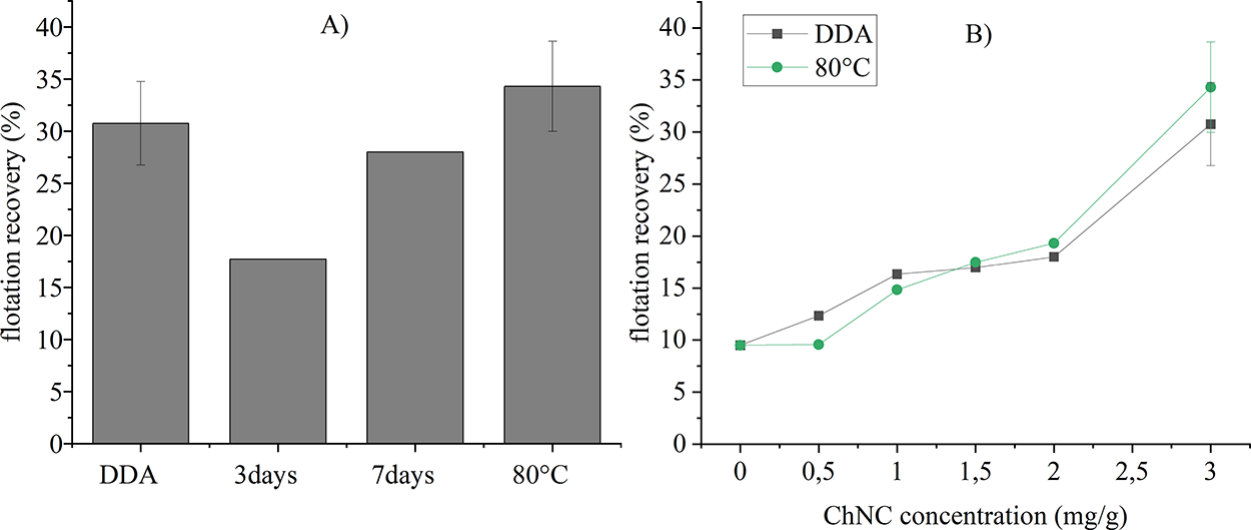
Flotation recovery of malachite using dodecyl amine (DDA) and *N*-decyl ChNCs. (A) DDA and functionalized Dec-ChNC under different reaction conditions at a concentration of 3 mg g^–1^; (B) DDA and Dec-ChNC reacted at 80 °C at different concentrations. The error bars represent standard deviations.

The flotation recovery of malachite using dodecyl amine (DDA) as a conventional collector and *N*-decyl ChNCs as an environmentally-friendly alternative is shown in Figure 6.

**Figure 7:**
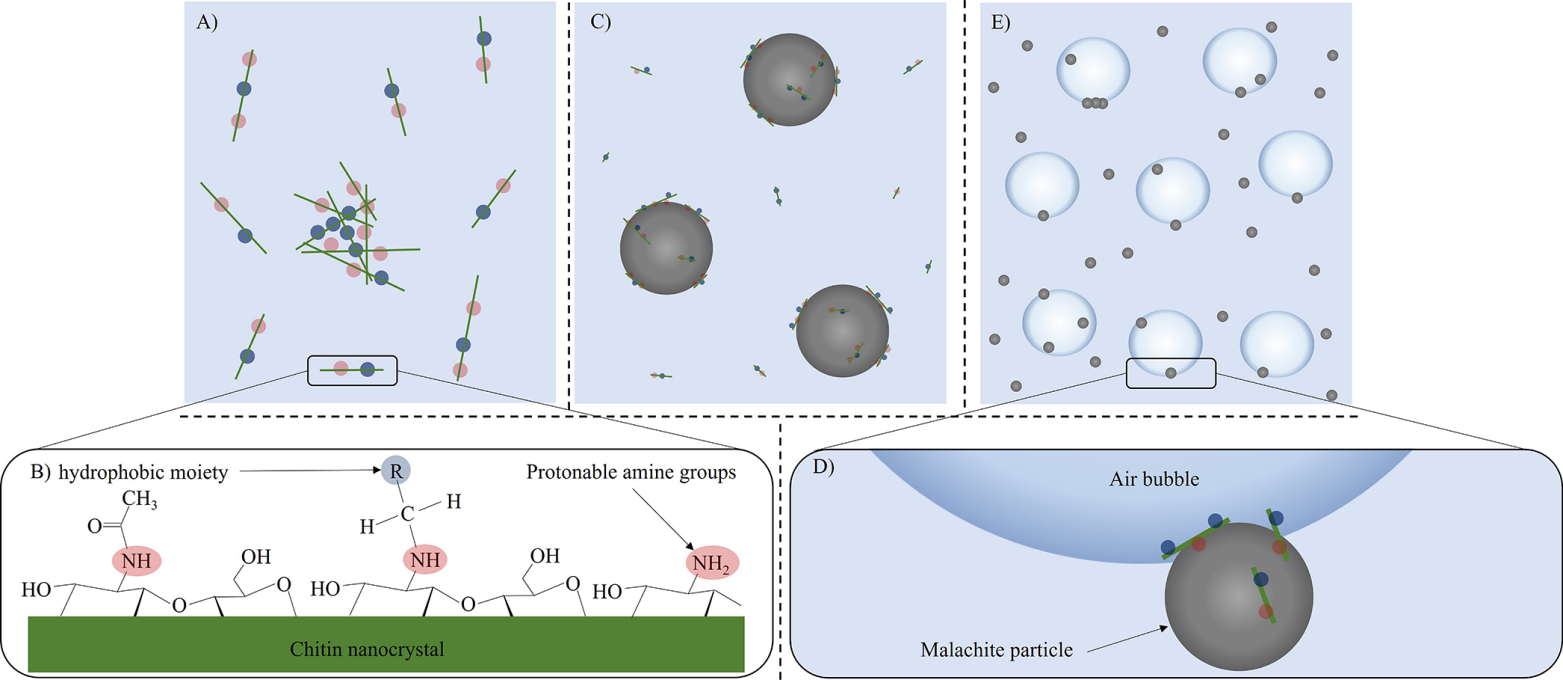
Behavior of functionalized ChNCs during the flotation process (not to scale): (A) dispersion of ChNCs in the pulp with partial aggregation; (B) functional groups on the chitin nanocrystal (ChNC) surface; (C) adsorption of positively charged ChNCs onto negatively charged malachite particles; (D) a ChCNC-coated malachite particle attached to an air bubble; and (E) the attachment of malachite particles on air bubbles during flotation.

The recovery of malachite was more efficient when ChNCs were functionalized for longer times or at higher temperatures. Within the experimental error, both *N*-decyl ChNCs functionalized for 7 days or at 80 °C gave recoveries comparable to DDA at a concentration of 3 mg g^–1^. As seen in Figure 6, the *N*-decyl ChNC sample produced at 80 °C showed the highest malachite recovery of all samples at 34%. A schematic summarizing the behavior of functionalized ChNCs during the flotation process is shown in Figure 7. The recovery of malachite with ChNC reagents proved that these bio-colloids can be properly functionalized to boost the interaction with mineral surfaces and render them sufficiently hydrophobic for separation in froth flotation.

## Conclusions

A novel, renewable flotation reagent for malachite was produced in a simple one-pot reaction of partially deacetylated chitin nanocrystals (ChNCs) with aldehydes in a reductive alkylation of the free amino groups. The functionalization with aliphatic aldehydes of different chain lengths under various conditions was tested, showing that temperature had the most significant influence on the degree of substitution. The incorporation of alkyl chains decreased the ζ-potential and increased the degree of hydrophobicity of ChNC films. The presence of the functionalized ChNCs caused a significant change of the ζ-potential of malachite, indicating their adhesion on the mineral surface under acidic aqueous conditions. The efficiency of *N*-alkylated ChNCs in the recovery of malachite was tested with a Hallimond tube and compared to the performance of dodecyl amine, showing that ChNCs led to slightly higher recoveries of malachite at relatively low concentrations compared to commercially available collectors. With these promising results, further optimization of ChNCs functionalization is of interest for future work. A higher degree of deacetylation would further boost the adhesion of ChNCs on the malachite surface and simultaneously allow the incorporation of higher concentrations of *N*-alkyl or other hydrophobic, organic functional groups on the ChNC surface. The presence of ChNCs may also affect other interfaces present in flotation systems, for instance, the gas–liquid interface in the slurry or froth phase, an aspect worth studying. In summary, this work demonstrates that a relatively simple functionalization route can be used for ChNCs to render them useful as froth flotation reagents, representing a viable renewable alternative to hitherto used fossil-based chemistries.

## Supporting Information Available

Chemical composition of chitin and chitosan; cumulative particle size distribution of malachite with different size quantiles and the Sauter mean diameter (*D*_3,2_); the ^13^C solid-state NMR spectra with integrals of the peaks from 12.5–34 to 53–107 ppm for native and *N*-decyl functionalized chitin nanocrystals; the scheme of the Hallimond tube used for flotation experiments; images of a *N*-ChNC sample stored in a fridge at 4 °C for a month and after ultrasound treatment using a dip sonicator; average size of *N*-decyl ChNCs functionalized for 3 h and 7 days (PDF)
