# Supplementary material for: N-Alkylated Chitin Nanocrystals as a Collector in Malachite Flotation
Source: ACS Sustain Chem Eng. 2022 Aug 4;10(32):10570–8. doi: 10.1021/acssuschemeng.2c01978 (PMC9382668; doi:10.1021/acssuschemeng.2c01978)
Supplement: Supplementary file 1 — sc2c01978_si_001.pdf [file sc2c01978_si_001.pdf]

# *N*-Alkylated Chitin Nanocrystals as Collector in Malachite Flotation

*Robert Hartmann*[a,b]*, *Marco Beaumont*[c], *Eva Pasquier*[d,e], *Thomas Rosenau*[c] and *Rodrigo Serna-Guerrero*[a]

[a] Department of Chemical and Metallurgical Engineering, School of Chemical Engineering, Aalto University, Finland, P.O. Box 12200, FIN-00076 Espoo, Finland

[b] Fraunhofer Center for Chemical-Biotechnological Processes, D-06237 Leuna, Germany

[c] Department of Chemistry, Institute for Chemistry of Renewable Resources, University of Natural Resources and Life Science, A-3430 Tulln, Austria

[d] Department of Bioproducts and Biosystems, School of Chemical Engineering, Aalto University, FIN-00076 Espoo, Finland

[e] Université Grenoble Alpes, CNRS, Grenoble INP (Institute of Engineering), LGP2, F-38000 Grenoble, France

(*corresponding author: Robert.Hartmann@igb.fraunhofer.de)

Number of pages: 8, Number of figures: 10, Number of Tables: 0

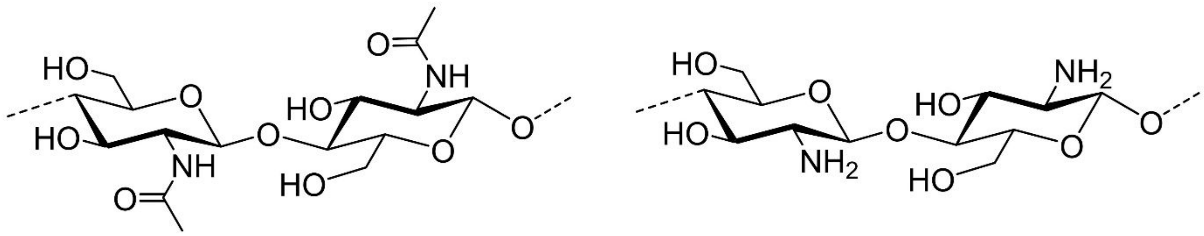


Figure S1: Chemical structure of chitin (left) and chitosan (right).

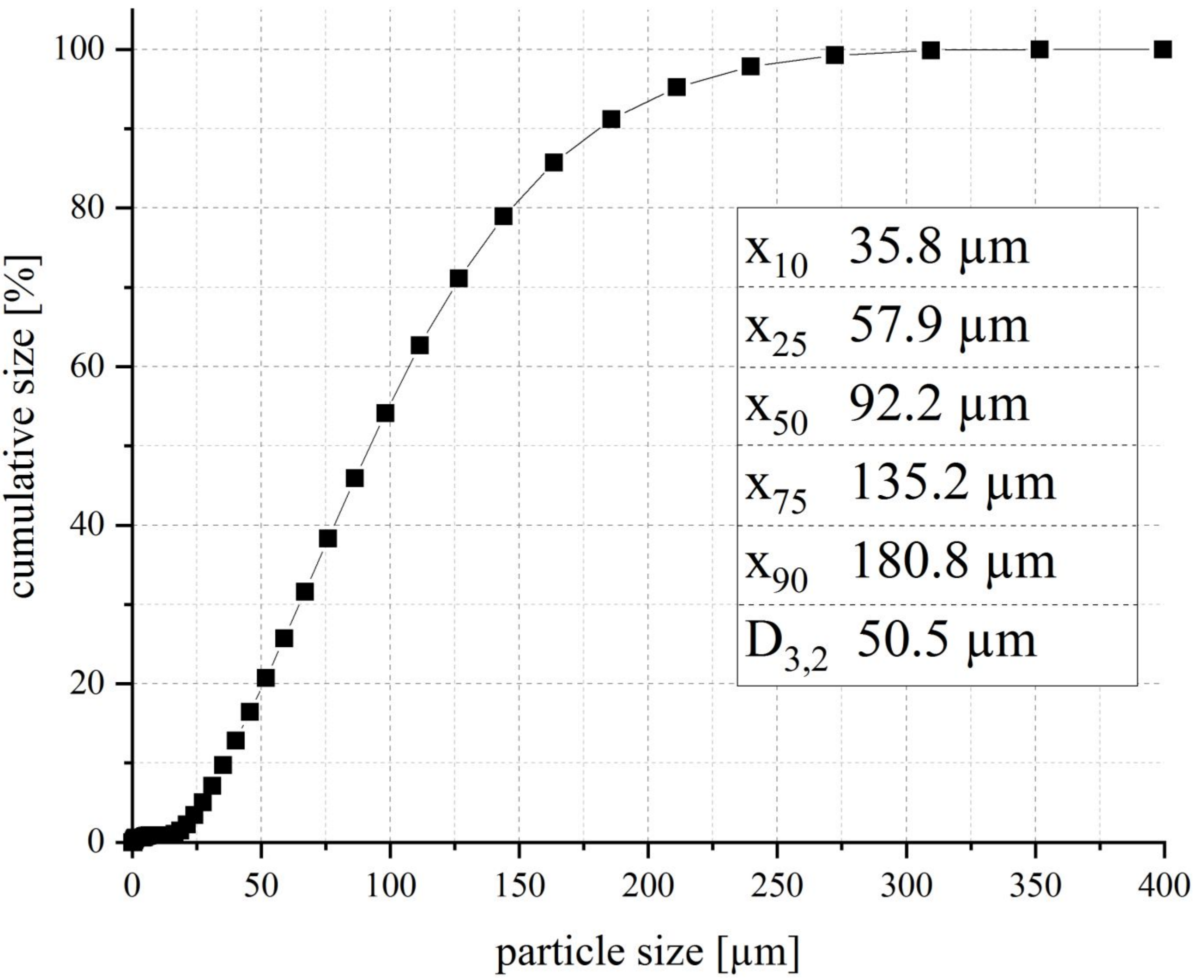


Figure S2: Cumulative particle size distribution of malachite with different size quantiles and the Sauter mean diameter ($D_{3,2}$).

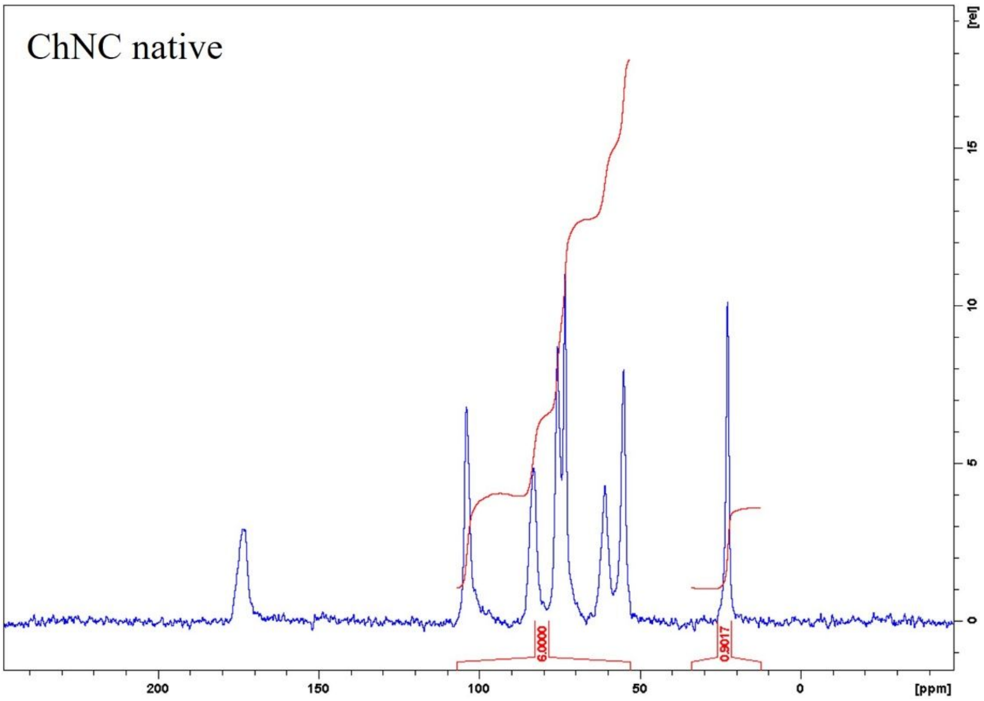


Figure S3: $^{13}C$ solid-state NMR spectra with integrals of the peaks from 12.5-34 ppm and 53-107 ppm for native chitin nanocrystals

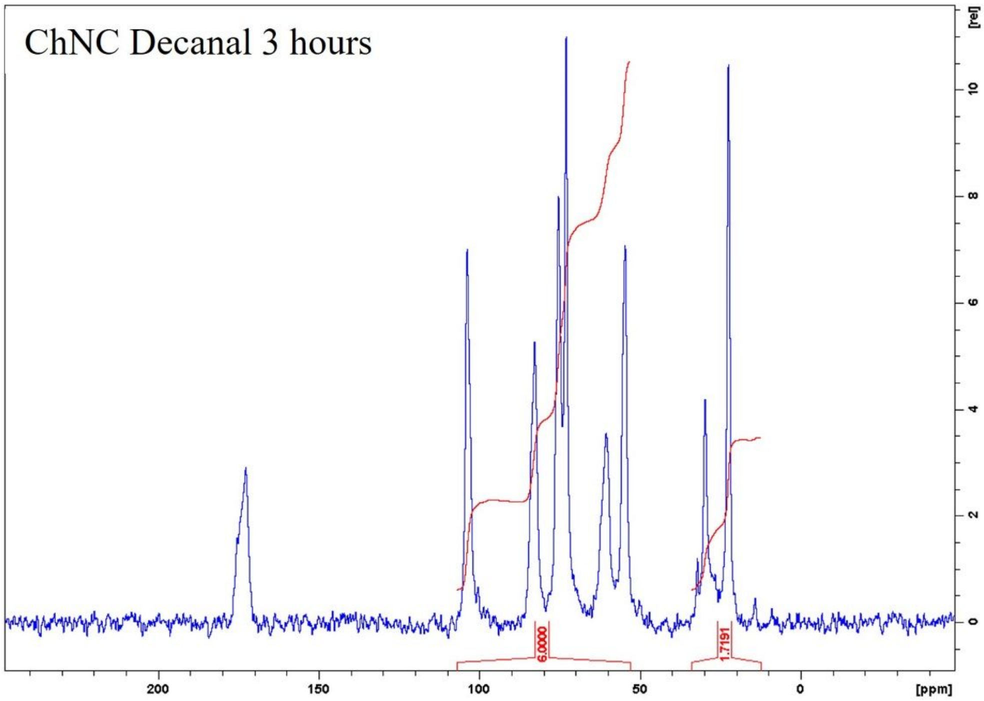


Figure S4: $^{13}C$ solid-state NMR spectra with integrals of the peaks from 12.5-34 ppm and 53-107 ppm for chitin reacted with decanal for 3 hours under ambient temperature.

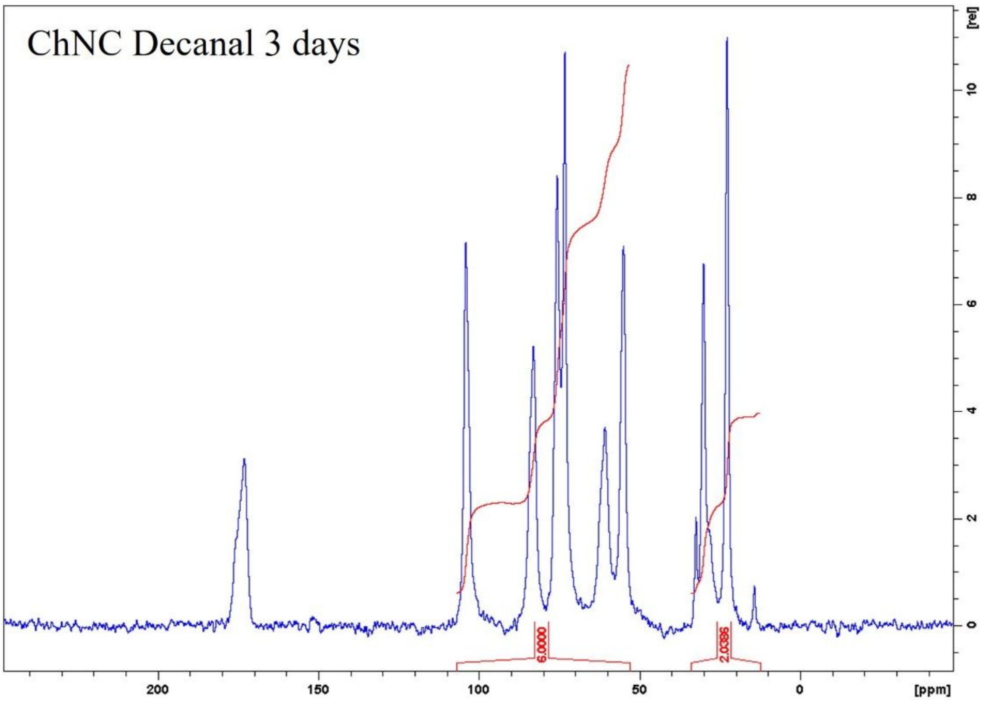


Figure S5: $^{13}C$ solid-state NMR spectra with integrals of the peaks from 12.5-34 ppm and 53-107 ppm for chitin reacted with decanal for 3 days under ambient temperature.

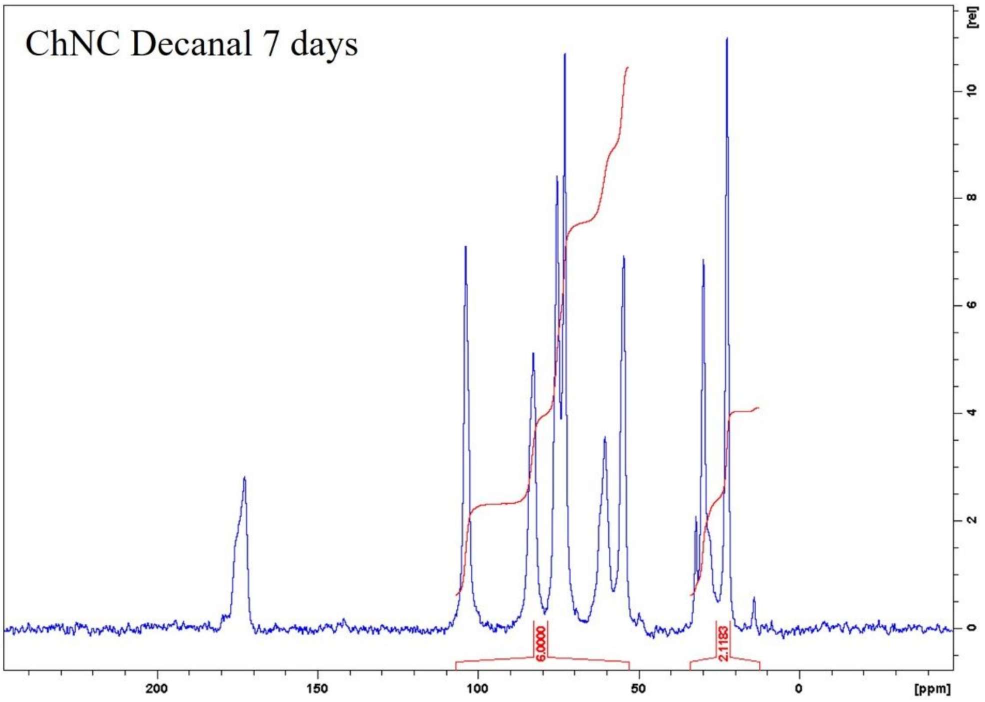


Figure S6: $^{13}C$ solid-state NMR spectra with integrals of the peaks from 12.5-34 ppm and 53-107 ppm for chitin reacted with decanal for 7 days under ambient temperature.

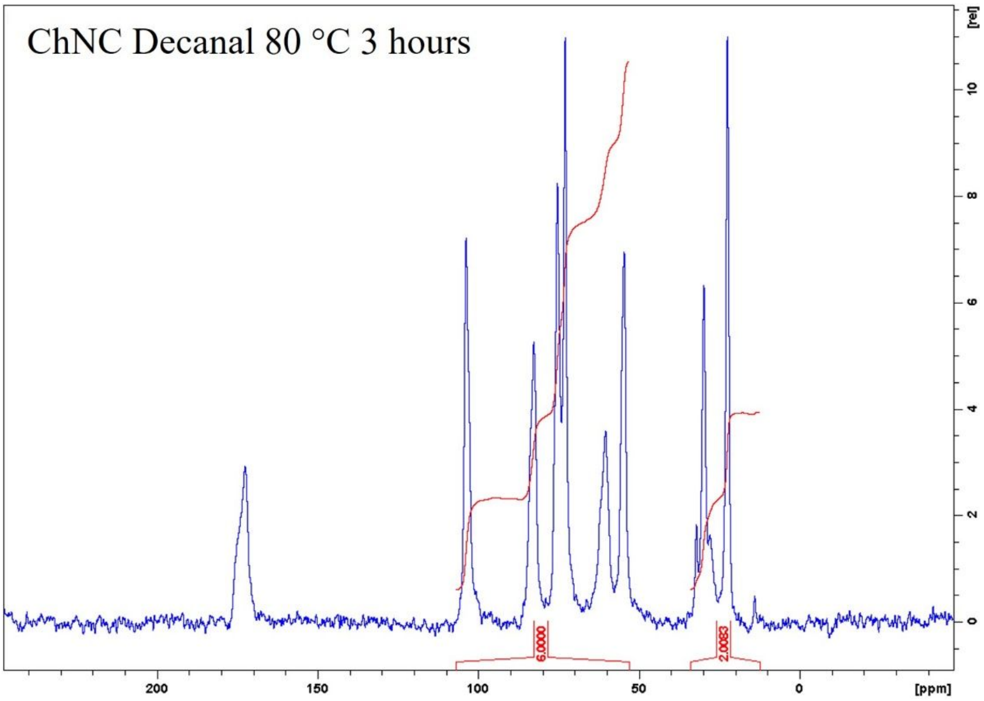


Figure S7: $^{13}C$ solid-state NMR spectra with integrals of the peaks from 12.5-34 ppm and 53-107 ppm for chitin reacted with decanal for 3 hours at 80°C.

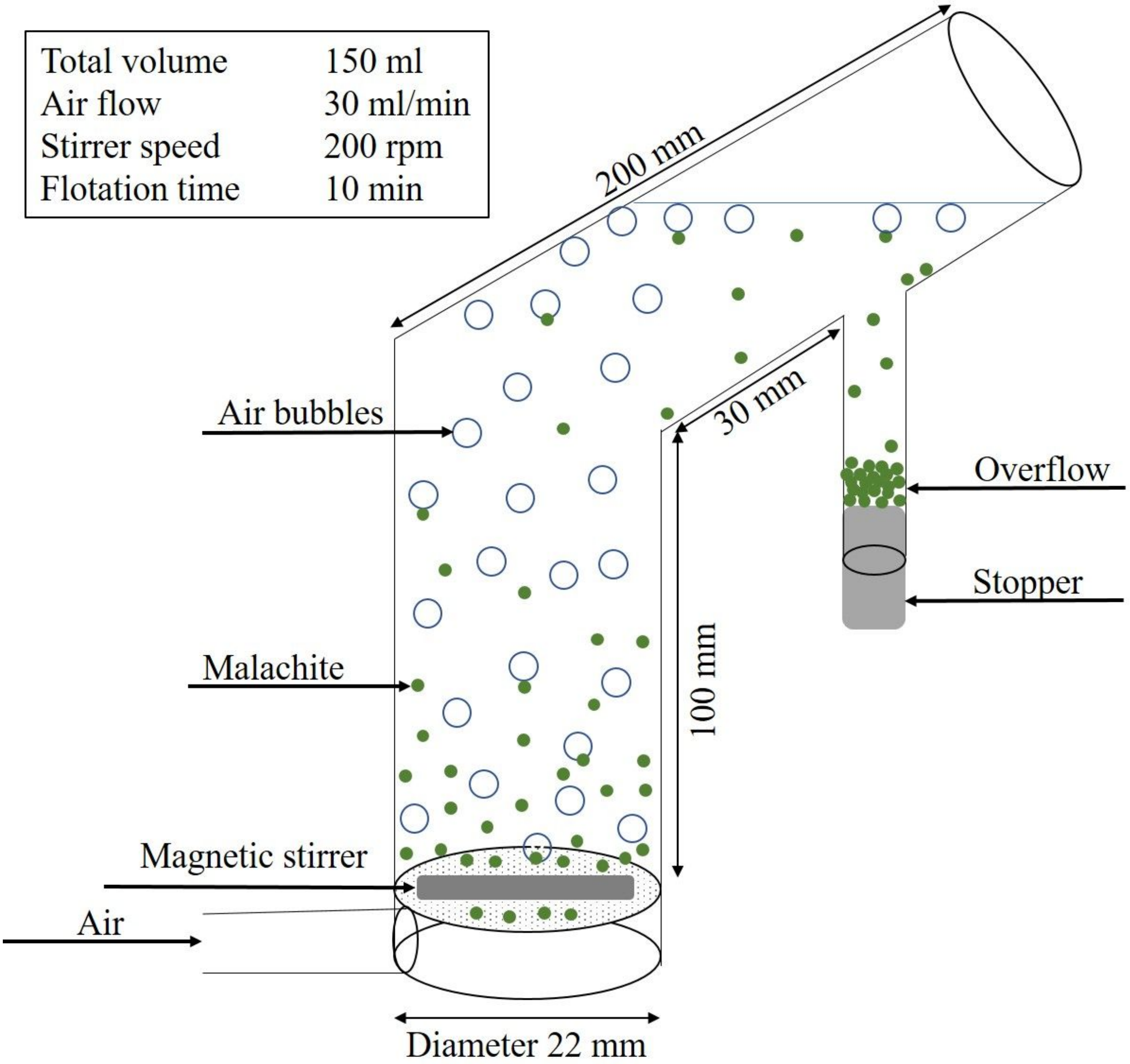


Figure S8: Scheme of the Hallimond tube used for malachite flotation.

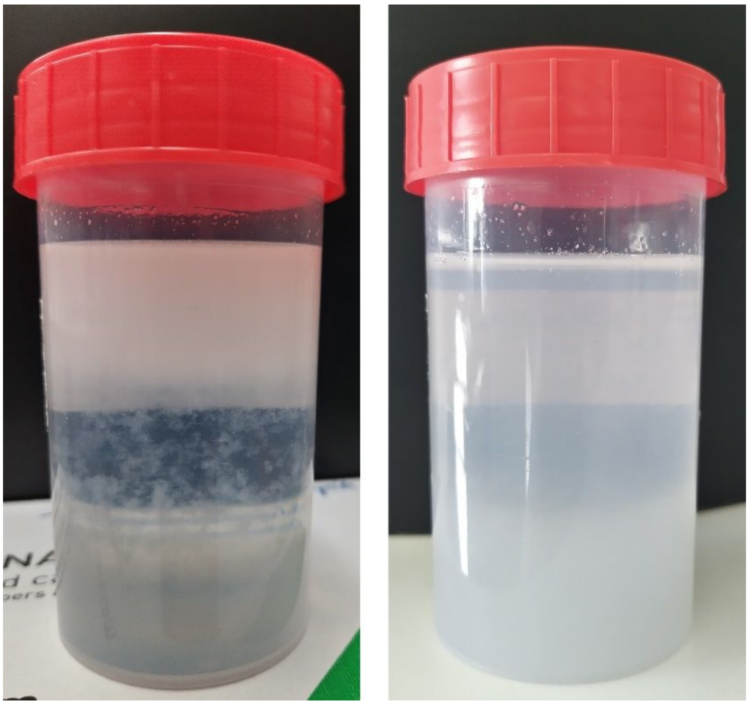

Figure S9: A ChNC sample functionalized with decanal being stored in a fridge at 4˚C for a month (left), and after ultrasound treatment using a dip sonicator (right).

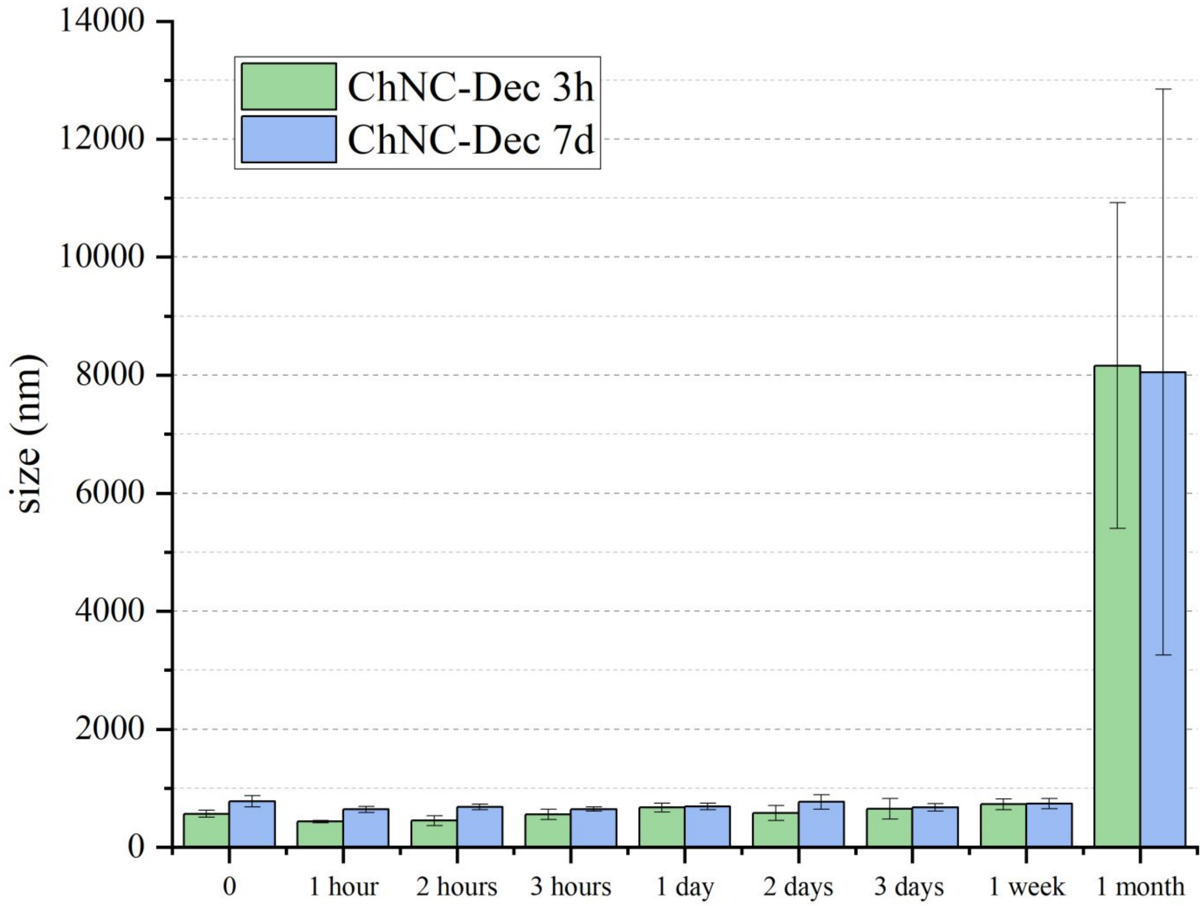


Figure S10: The average size of ChNCs functionalized with decanal reacted for 3 hours and 7 days as a function of time.
